# Phosphatase PPM1A negatively regulates P-TEFb function in resting CD4^+^ T cells and inhibits HIV-1 gene expression

**DOI:** 10.1186/1742-4690-9-52

**Published:** 2012-06-22

**Authors:** Sona Budhiraja, Rajesh Ramakrishnan, Andrew P Rice

**Affiliations:** 1 Department of Molecular Virology and Microbiology, Baylor College of Medicine, Houston, Texas, 77030, USA

**Keywords:** P-TEFb, HIV-1, CDK9, PPM1A

## Abstract

**Background:**

Processive elongation of the integrated HIV-1 provirus is dependent on recruitment of P-TEFb by the viral Tat protein to the viral TAR RNA element. P-TEFb kinase activity requires phosphorylation of Thr186 in the T-loop of the CDK9 subunit. In resting CD4^+^T cells, low levels of T-loop phosphorylated CDK9 are found, which increase significantly upon activation. This suggests that the phosphorylation status of the T-loop is actively regulated through the concerted actions of cellular proteins such as Ser/Thr phosphatases. We investigated the role of phosphatase PPM1A in regulating CDK9 T-loop phosphorylation and its effect on HIV-1 proviral transcription.

**Results:**

We found that overexpression of PPM1A inhibits HIV-1 gene expression during viral infection and this required PPM1A catalytic function. Using an artificial CDK tethering system, we further found that PPM1A inhibits CDK9, but not CDK8 mediated activation of the HIV-1 LTR. SiRNA depletion of PPM1A in resting CD4^+^T cells increased the level of CDK9 T-loop phosphorylation and enhanced HIV-1 gene expression. We also observed that PPM1A protein levels are relatively high in resting CD4^+^T cells and are not up-regulated upon T cell activation.

**Conclusions:**

Our results establish a functional link between HIV-1 replication and modulation of CDK9 T-loop phosphorylation by PPM1A. PPM1A represses HIV-1 gene expression by inhibiting CDK9 T-loop phosphorylation, thus reducing the amount of active P-TEFb available for recruitment to the viral LTR. We also infer that PPM1A enzymatic activity in resting and activated CD4^+^ T cells are likely regulated by as yet undefined factors.

## Background

Recent genome-wide studies have revealed that 20-30% of mammalian genes are occupied by RNAPII paused at promoter proximal regions and require P-TEFb for the transition into productive elongation [1]. Core P-TEFb is composed of CDK9 as a catalytic subunit and a regulatory Cyclin subunit, either Cyclin T1 or Cyclin T2 [2]. Cyclin T1 is the predominant regulatory subunit of P-TEFb in the majority of mammalian tissues examined so far [3]. Two isoforms of CDK9 are expressed, a major 42 kDa protein and a minor 55 kDa protein that arises from an upstream transcriptional start site [4,5]. Like other CDK family members, CDK9 contains a T-loop structure which blocks the catalytic cleft of the enzyme in the absence of Cyclin T1. Upon Cyclin T1 binding, the T-loop is repositioned which facilitates unblocking of the catalytic core and the phosphorylation of the Thr186 residue thereby permitting access of substrates and ATP to the catalytic core of the enzyme. [6]. In addition to its central role in regulating cellular gene expression, P-TEFb is also required for replication of Human Immunodeficiency Virus-1 (HIV-1) [7]. RNAPII is able to initiate transcription from the viral 5’ LTR at a relatively high basal rate, but the transition to processive elongation is inhibited by the action of two negative elongation factors, DSIF and NELF [8]. To overcome this block to processive elongation, Tat binds Cyclin T1 through a Tat-Recognition Motif (TRM) in the amino terminus of Cyclin T1 and recruits P-TEFb to the TAR RNA element that forms at the 5’ end of all viral transcripts [9]. CDK9 is then able to phosphorylate the carboxyl terminal domain (CTD) of the paused RNAPII and subunits of DSIF and NELF, thereby stimulating processive elongation [2,3,7].

P-TEFb kinase activity is dependent on association with a Cyclin subunit and phosphorylation of a conserved Thr186 residue in the CDK9 T-loop. The T-loop phosphorylation status of CDK9 determines the biological activity and may affect the sub-nuclear localization of P-TEFb [10,11]. Notably, P-TEFb that is phosphorylated on the CDK9 Thr186 residue is sequestered within the 7SK RNA-HEXIM1 complex, where its kinase activity is inhibited due to association with the HEXIM1 protein [12]. Given that P-TEFb-dependent transcriptional elongation is an abundantly exploited step in regulating cellular gene expression, it is not surprising that P-TEFb kinase activity is stringently regulated to prevent any inadvertent activation of genes. Association of the kinase-active P-TEFb with the 7SK snRNA-HEXIM1 may provide a reservoir of pre-activated P-TEFb, from which cellular or viral transcription factors can extract it and selectively activate gene expression from their cognate promoters [2,13]. The HIV-1 protein Tat has been shown to not only compete with HEXIM1 for binding to the activated P-TEFb complex, but it also extracts the enzymatically active but sequestered P-TEFb from the 7SK snRNA-HEXIM1 complex [14,15]. In addition to recruiting P-TEFb, Tat was recently demonstrated to enlist additional elongation factors, such as ELL2, AFF4, ENL and AF9, leading to the formation of a Super Elongation Complex at the HIV-1 promoter [16,17].

Resting CD4^+^T cells are non-permissive for HIV-1 replication and the level of CDK9 T-loop phosphorylation is very low in these cells. However, upon T cell activation there is a rapid increase in T-loop phosphorylation which does not require protein synthesis, and this coincides with increased permissivity for HIV-1 replication [18]. It is likely that the status of CDK9 T-loop phosphorylation in resting and activated CD4^+^T cells is the result of an interplay between kinases and phosphatases. Although CDK9 has been reported to be autophosphorylated in *in vitro* assays, it is uncertain if this process occurs efficiently *in vivo* or if CDK9 is phosphorylated by an activating kinase [19]. CDK7, a metazoan CAK (CDK-Activating Kinase) that activates CDKs involved in cell cycle control and is also part of the transcription factor TFIIH, has been suggested to be a CDK9-Activating Kinase [20]. However, attempts to demonstrate that CDK7 can phosphorylate the CDK9 T-loop in vitro have thus far been unsuccessful [12,21].

In contrast to the ambiguity regarding the mode of CDK9 T-loop phosphorylation, phosphatases have been identified that can dephosphorylate the T-loop. Phosphatases belonging to the PPP family such as PP1α and PP2B have been shown to co-operatively dephosphorylate CDK9 in response to signals of stress and this releases core P-TEFb from the inhibitory 7SKsnRNA-HEXIM1 complex [22]. We reported that the Mg^2+^/Mn^2+^-dependent monomeric phosphatase PPM1A associates with CDK9 *in vivo* as determined by co-immunoprecipitation. PPM1A can dephosphorylate the T-loop *in vitro* in both the core and 7SK snRNP P-TEFb complexes, and depletion of PPM1A in HeLa cells resulted in an increase in the total level of CDK9 T-loop phosphorylation [23].

In this study, we investigated the roles of the phosphatase PPM1A in regulating CDK9 phosphorylation and HIV-1 replication. We found that overexpression of PPM1A inhibits HIV-1 infection and gene expression. Furthermore, utilizing an artificial CDK tethering system [24,25], we show that suppression of HIV-1 transcription is due to selective inhibition of CDK9 by PPM1A, as the CDK8 kinase, part of the mediator complex involved in transcriptional initiation [26], was not inhibited by PPM1A in this system. We also show that depletion of PPM1A in primary resting CD4^+^T cells increases CDK9 T-loop phosphorylation, which also caused a concomitant augmentation of HIV-1 gene expression in these cells. Lastly, the protein level of PPM1A did not differ between resting and activated CD4^+^T cells, suggesting that the enzymatic activity of this protein is likely regulated through mechanisms that are not dependent upon fluctuations in its protein levels.

## Results

### Effect of PPM1A on HIV-1 infection and gene expression

**Figure 1:**
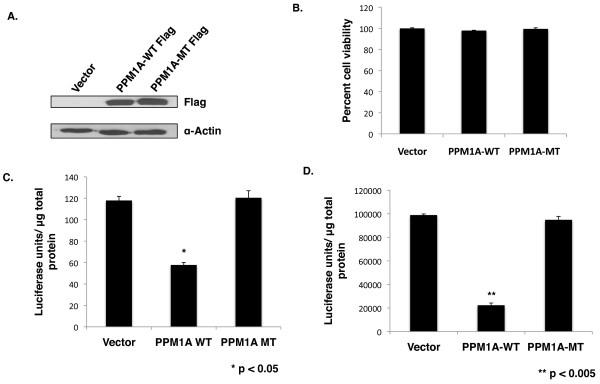
**PPM1A inhibits HIV-1 infection and gene expression. A.** HeLa cells were transfected with Flag-PPM1A-WT, Flag-PPM1A-MT, or empty vector expression plasmids. Cell lysates were harvested 48 hours after transfection and probed in an immunoblot for Flag expression and α-Actin. **B.** Triplicate HeLa cell cultures were transfected with PPM1A-WT, PPM1A-MT, or empty vector expression plasmids. Viability of the transfected cells was measured 48 hours after transfection using the Vi-CELL automatic cell analyzer. **C.** Triplicate HeLa cell cultures were transfected with PPM1A-WT, PPM1A-MT, or vector expression plasmids; cells were infected with a VSV-G pseudotyped HIV-1 NL4-3 Luciferase reporter virus at 24 hours post-transfection. Luciferase expression was measured in cells extracts at 24 hours post-infection and normalized to total protein in extracts. (* represents p value of < 0.05 in a paired *t*-test.) **D.** Triplicate HeLa cell cultures were co-transfected with pNL4-3 Luciferase proviral plasmid plus vector control or expression plasmids for PPM1A-WT or PPM1A-MT. Luciferase expression in cell extracts was measured at 48 hours post-transfection and was normalized to total protein in extracts. (**denotes p value of < 0.005 in a paired *t*-test.).

We previously reported that shRNA depletion of PPM1A in HeLa cells increases CDK9 T-loop phosphorylation approximately 2.5-fold *in vivo*, and PPM1A can dephosphorylate the T-loop *in vitro* in either the core or 7SK snRNP P-TEFb complex [23]. In this study, we therefore wanted to examine the effect of PPM1A overexpression on HIV-1 infection and gene expression. We validated the equal expression of the Flag tagged wild type (WT) PPM1A and the catalytically inactive mutant (MT) PPM1A R174G plasmids in HeLa cells (Figure 1A). We also characterized the effect of these plasmids on HeLa cell viability. HeLa cells were transfected with WT PPM1A, MT PPM1A or an empty vector plasmid and cell viability was determined using a Vi-Cell analyzer 48 hours after transfection. There was no difference in viability of cells transfected with the WT or the MT PPM1A plasmids compared to the cells transfected with empty vector control plasmid (Figure 1B).

To determine the effect of PPM1A on HIV-1 infection during the course of a single replication cycle, HeLa cells were transfected with a WT PPM1A expression plasmid, MT PPM1A R174G expression plasmid, or an empty vector plasmid. Cells were infected with a VSV-G pseudotyped HIV-1 NL4-3 Luciferase reporter virus at 24 hours post-transfection and Luciferase expression was measured at 24 hours post- infection (Figure 1C). In cells transfected with the WT PPM1A expression plasmid, there was a ~50% reduction in Luciferase expression relative to cells transfected with the vector control. There was no inhibition of the reporter virus in cells transfected with the MT PPM1A expression plasmid, indicating that PPM1A catalytic function is required for the inhibitory effect. The experiment shown in Figure 1C is representative of several independent replicate experiments.

To assess whether the inhibitory effect of PPM1A on the reporter virus acts on viral gene expression and is not restricted to an earlier point in the viral life cycle, we evaluated the effects of overexpression of PPM1A on a transfected HIV-1 proviral plasmid. HeLa cells were co-transfected with a HIV-1 NL4-3 proviral luciferase reporter plasmid and a WT or MT R174G PPM1A expression plasmid, as well as an empty vector plasmid. Cell extracts were prepared 48 hours post-transfection and assayed for luciferase expression (Figure 1D). In cells transfected with the WT PPM1A plasmid, there was a ~80% reduction in proviral gene expression relative to cells that were transfected with the empty vector. In cells transfected with the MT PPM1A, there was a no reduction in proviral gene expression relative to control cells, again indicating that the inhibition of proviral gene expression is dependent upon PPM1A catalytic activity. The experiment shown in Figure 1D is representative of several independent replicate experiments.

### Overexpression of PPM1A inhibits CDK9 but not CDK8 activation of reporter plasmid

**Figure 2:**
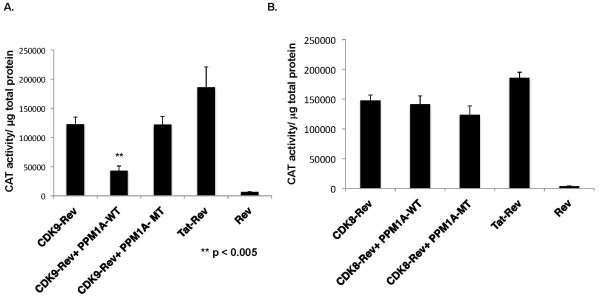
**PPM1A dephosphorylates CDK9, but not CDK8 in an artificial CDK tethering system.** Triplicate HeLa cell cultures were transfected with pSLIIB-CAT reporter plasmid and expression plasmids for different effector proteins: CDK9-Rev, CDK8-Rev, PPM1A-WT, PPMIA-MT, Tat-Rev, and Rev as indicated. Cell extracts were prepared at 48 hours post-transfection and CAT activity was measured and normalized to total protein in extracts. (**denotes p value of < 0.005 in a paired *t*-test.).

To evaluate the specificity of PPM1A in the inhibition of CDK9 function *in vivo*, we used an artificial CDK tethering system based upon a HIV-1 LTR reporter plasmid termed pSLIIB-CAT in which the TAR RNA element is replaced by the Rev-Response Element (RRE) [24]. We have previously shown that CDK9-Rev and CDK8-Rev fusion proteins activate this reporter plasmid [27,28]. The pSLIIB-CAT plasmid was co-transfected in HeLa cells with CDK9-Rev or CDK8-Rev fusion proteins expression plasmids, plus a WT or MT (R174G) PPM1A expression plasmid. Tat-Rev fusion protein and Rev expression plasmids were also co-transfected to serve as a positive and negative control, respectively. The CDK9-Rev and Tat-Rev proteins activated the reporter plasmid 19-fold and 29-fold, respectively, relative to cells transfected with Rev protein (Figure 2A). Data shown are the average of two independent experiments. Co-transfection of the WT PPM1A expression plasmid reduced CDK9-Rev activation of the reporter plasmid by ~65%, while the MT PPM1A protein did not have any effect on CDK9-Rev activation of the reporter. The CDK8-Rev fusion protein activated the reporter plasmid ~40-fold (Figure 2B). Data shown are representative of several independent experiments. In contrast to the inhibition of the CDK9-Rev protein, co-transfection of the WT or the MT PPM1A plasmid had no significant effect on CDK8-Rev activation of the reporter plasmid. These data suggest that PPM1A negatively regulates CDK9 but not CDK8 *in vivo*. Based on the data presented in Figures 1 and 2, we conclude that inhibition of CDK9 function by PPM1A overexpression is likely the result of inhibition of T-loop dephosphorylation, as we have previously shown that PPM1A dephosphorylates the CDK9 T-loop *in vitro* and regulates the level of T-loop phosphorylation *in vivo*. Furthermore, the negative regulation of CDK9 phosphorylation by PPM1A results in inhibition of HIV-1 gene expression. Repression of the HIV-1 LTR reporter plasmid in the CDK tethering system suggests that PPM1A inhibition of viral LTR-directed gene expression is largely the consequence of inhibition of CDK9 function and not inhibition of other transcription factors such as NF-κB or NFAT.

### SiRNA depletion of PPM1A in primary resting CD4^+^T cells increases CDK9 T-loop phosphorylation

**Figure 3:**
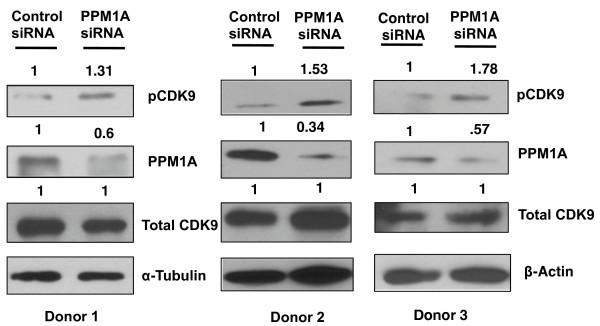
**SiRNA depletion of PPM1A increases CDK9 T-loop phosphorylation in resting CD4**^**+**^**T cells.** Resting CD4^+^T cells from three healthy blood donors were nucleofected with siRNA against PPM1A or control siRNA on two consecutive days. Cell extracts were prepared 48 hours post-Nucleofection and evaluated for PPM1A, T-loop phosphorylated CDK9 (pCDK9), total CDK9, α-Tubulin and β-Actin (loading control) in immunoblots. Protein levels shown on top of panels were quantified by normalizing to α-Tubulin or β-Actin and set at 1.0 in control siRNA-transfected cells.

We have previously observed that CDK9 T-loop phosphorylation is low in resting CD4^+^ T cells isolated from healthy blood donors [18]. The mechanisms involved in regulating this low level of phosphorylation are unknown. To test if PPM1A may play a role in repressing T-loop phosphorylation, resting CD4^+^T cells obtained by negative selection from three healthy blood donors were nucleofected with negative-control siRNAs or siRNAs against PPM1A on two consecutive days. Cell lysates were prepared 48 hours post-transfection and evaluated for expression levels of PPM1A, T-loop phosphorylated CDK9 and total CDK9 levels (Figure 3). In donor 1, PPM1A-specific siRNAs reduced PPM1A protein levels by 40%; T-loop phosphorylation increased 1.31-fold and total CDK9 levels remained unchanged. In donor 2, where PPM1A-specific siRNAs reduced PPM1A protein levels 66%, there was a corresponding 1.53-fold increase in T-loop phosphorylated CDK9, while total CDK9 levels were unchanged. In donor 3, PPM1A-specific siRNAs reduced PPM1A by ~40% and there was a 1.78-fold increase in T-loop phosphorylated CDK9 relative to control siRNA treated cells. The enhancement of CDK9 T-loop phosphorylation by depletions of PPM1A in three donors indicates that this phosphatase negatively regulates P-TEFb function in resting CD4^+^ T cells.

### PPM1A depletion in resting CD4^+^T cells increases HIV-1 gene expression

**Figure 4:**
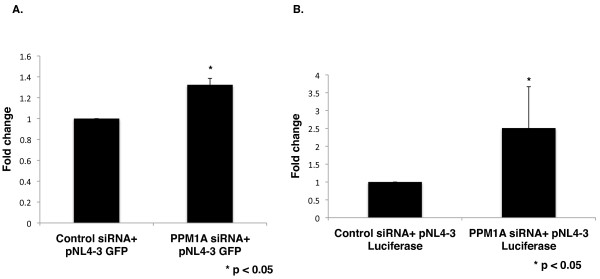
**PPM1A knockdown in resting CD4**^**+**^**T cells increases HIV-1 gene expression. A**. Resting CD4^+^T cells isolated from three healthy donors were nucleofected with control siRNA or siRNA against PPM1A plus a HIV-1 proviral reporter plasmid which expresses GFP. GFP expression was analysed 48 hours post-transfection. Fold-change in percentage of GFP+ cells was calculated by setting the GFP values to 1.0 in control siRNA treated cells. **B**. Resting CD4^+^T cells from five healthy donors were nucleofected similar to A, except that they were nucleofected with a HIV-1 proviral reporter plasmid that expresses luciferase. Luciferase expression was analyzed 48 hours post-transfection and normalized to total cellular protein. Fold-change in luciferase expression was calculated by setting the luciferase values to 1.0 in control siRNA treated cells.

The restrictive nature of resting CD4^+^T cells to productive HIV-1 replication is a result of multiple factors, including the limited availability of catalytically active P-TEFb. Since our results indicate that PPM1A depletion increases levels CDK9 T-loop phosphorylation in resting CD4^+^T cells, we wanted to determine if this would increase HIV-1 gene expression. Resting CD4^+^T cells were isolated from healthy blood donors by negative selection and nucleofected with control siRNAs or siRNAs against PPM1A and a HIV-1 proviral reporter plasmid that expresses a GFP or a Luciferase reporter protein. We analyzed three donors for the GFP reporter and five donors with the Luciferase reporter. GFP expression was examined at 48 hours post-transfection by flow cytometry (Figure 4A). Depletion of PPM1A led to a statistically significant 30% increase in the percentage of GFP positive cells compared to control siRNA treated cells. Similarly in PPM1A depleted cells nucleofected with a HIV-1 proviral reporter plasmid that expresses Luciferase, there was a statistically significant 2.54-fold increase in Luciferase gene expression compared to control siRNA treated cells (Figure 4B). These results indicate that by dephosphorylating the CDK9 T-loop in resting CD4^+^T cells, PPM1A likely contributes to the restrictive environment for HIV-1 replication in these cells.

### PPM1A is expressed at high levels in resting CD4^+^T cells

**Figure 5:**
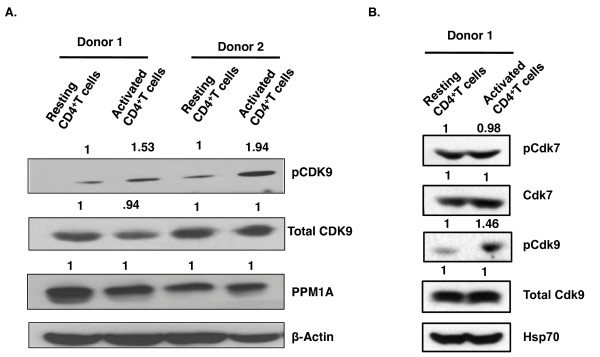
**PPM1A is expressed at high levels in resting CD4**^**+**^**T cells.** Resting CD4^+^T-cells isolated by negative selection from two healthy donors were activated with anti-CD3/CD28 Dynabeads. Cell lysates were prepared from resting CD4^+^T cells and cells activated for 48 hours. Immunoblots were performed to measure levels of: **A.** total CDK9, T-loop phosphorylated CDK9 (pCDK9), PPM1A and β-Actin (loading control) and **B.** total CDK9 and CDK7, T-loop phosphorylated CDK9 (pCDK9) and CDK7 (pCDK7), and HSP-70 (loading control). Protein levels shown on top of panels were quantified by normalizing to β-Actin or Hsp70 and set at 1.0 in resting CD4^+^T cells.

Given our results that depletion of PPM1A in resting CD4^+^T cells increased T-loop phosphorylated CDK9 levels, it is likely that high levels of PPM1A can contribute to repression of T-loop phosphorylation in resting CD4^+^T cells. Therefore, we wished to assess the protein levels of PPM1A, T-loop phosphorylated CDK9 and total CDK9 in resting and activated CD4^+^T cells. Because CDK7 may be an upstream activating kinase for CDK9, we also examined the levels of T-loop phosphorylated CDK7 (pCDK7) and total CDK7 in resting and activated CD4^+^T cells. Resting CD4^+^T cells purified by negative selection from two healthy blood donors were activated with αCD3/CD28 Dynabeads for 48 hours and cell lysates prepared for immunoblot analysis (Figure 5). As expected, the levels of CDK9 T-loop phosphorylation were up-regulated upon activation of resting CD4^+^T cells. In contrast, PPM1A was expressed at high levels in resting CD4^+^T cells and its levels were unaffected by activation in both the donors (Figure 5A). The levels of total CDK7 and pCDK7 were also unchanged between resting and activated CD4^+^T cells in donor 1 (Figure 5B). Similar results were obtained for another donor as well (data not shown). These results suggest that PPM1A and total CDK7 and pCDK7 protein levels do not differ between resting and activated CD4^+^T cells. It is probable that PPM1A catalytic function is modulated through association with as yet uncharacterized regulatory partners or post-translational modifications, and these modulations may result in a large net increase in CDK9 T-loop phosphorylation following T cell activation.

## Discussion

Post-translational modifications of P-TEFb subunits by ubiquitination, acetylation, and phosphorylation regulate its kinase activity [29]. While these modifications play important roles in modulation of P-TEFb function, P-TEFb kinase activity is strictly dependent on phosphorylation of Thr186 in the CDK9 T-loop [30]. Therefore, by modulating the phosphorylation status of the T-loop, cellular phosphatases or kinases can link productive transcriptional elongation to extracellular signals such as T cell activation. The role of PPM1A in regulating cellular signalling pathways important for growth and stress response is well characterized [31,32]. Furthermore, dysregulation of PPM1A function could have important implications for the cellular processes that it regulates, exemplified by a recent study which assessed the role of PPM1A *in vivo* using a mouse knockout model [33]. The PPM1A-deficient mice did not exhibit developmental abnormalities but were defective in keratinocyte-mediated re-epithelization due to impaired PPM1A regulated suppression of Smad2 signalling. The lack of developmental abnormalities suggests that there might be functional redundancies between PPM family members, PPM1A and PPM1B.

We assessed the role of PPM1A in regulating CDK9 T-loop phosphorylation in resting CD4^+^T cells, the major physiological reservoir for latent HIV-1 [34]. Our results demonstrate that PPM1A is a negative regulator of CDK9 T-loop phosphorylation in resting CD4^+^T cells. Although we observed a modest increase in CDK9 phosphorylation by siRNA depletion of PPM1A in resting CD4^+^T cells relative to the effect in HeLa cells (~1.5-fold Figure 4 vs. ~2.5-fold Figure 4 ref [23]), the effect is likely to be significant given that quiescent CD4^+^T lymphocytes are difficult to transfect with siRNAs and are metabolically inactive and carry out low levels of protein synthesis.

Regulation of CDK9 T-loop phosphorylation by cellular effectors has important implications for HIV-1 replication since productive proviral transcription is dependent on recruitment of T-loop phosphorylated P-TEFb to the viral promoter. We have provided multiple lines of evidence which indicate that PPM1A represses HIV-1 replication in HeLa cells and resting CD4^+^T cells. Based upon our observation that PPM1A depletion in resting CD4^+^T cells leads to a statistically significant increase in HIV-1 gene expression, it is likely that PPM1A acts at the transcriptional level in these cells to inhibit HIV-1 replication. However it is now well accepted that multiple transcriptional and post-transcriptional blocks contribute to a restrictive environment for HIV-1 replication in quiescent CD4^+^T cells [34]. Therefore in order to observe a considerable increase in HIV-1 replication in resting CD4^+^T cells, it will likely be necessary to overcome more than a single replication block such as that imposed by PPM1A.

In HeLa cells, overexpression of PPM1A inhibited infection of a HIV-1 reporter virus and also suppressed gene expression of a HIV-1 reporter plasmid. PPM1A also inhibited CDK9-mediated but not CDK8-mediated activation of the HIV LTR in an artificial CDK tethering system. PPM1A has been documented to be an IKK-β phosphatase that down-regulates TNFα mediated activation of NFκB [35]. While the cumulative results of our study indicate that the inhibition of HIV-1 gene expression is chiefly mediated through CDK9 dephosphorylation, it is possible that termination of NF-κB activation by PPM1A also plays a part in this inhibition. CDK9 and CDK8 exhibit differential specificity for the heptad repeats within the CTD of RNAPII, which has been attributed to differences in their respective activation segments [36]. The conserved Thr residue in the CDK9 T-loop is substituted by aspartic acid in CDK8 [36]. It is therefore tempting to speculate that this distinction in the active site conformations of CDK9 and CDK8 could be the determinant for the varying substrate specificity displayed by PPM1A in the heterologous CDK tethering system.

In contrast to our data, another Ser/Thr phosphatase PP1 has been shown to activate HIV-1 gene expression [37]. The distinct effects of PPM1A and PP1 on HIV-1 transcription might be attributed to the difference in their relative activities for the CDK9 T-loop and/or ability to modify other residues besides Thr186 in the CDK9 T-loop. PP1 has been shown to dephosphorylate Thr186, as well as Ser175 [38]. The dephosphorylation of Ser175 was implicated in activating CDK9 kinase activity and possibly upregulating HIV-1 transcription. While we have not determined the effect of PPM1A on Ser175, it has been previously reported that CDK9 enzymatic activity and its association with the 7SKRNA-HEXIM1 complex is dictated largely by Thr186 and not Ser175 phosphorylation [39].

Since CDK9 T-loop phosphorylation levels are low in resting CD4^+^T cells, it appears that the activity of PPM1A for the T-loop might be dominant over the uncharacterized mechanism by which the CDK9 T-loop is phosphorylated. Upon T cell activation, this appears to be reversed and the process by which CDK9 T-loop is phosphorylated likely overrides the mechanism by which it is dephosphorylated. As we have found that PPM1A and CDK7 (and T-loop phosphorylated CDK7) protein levels do not vary significantly between resting and activated CD4^+^T cells, changes in protein levels for these enzymes do not account for the differences in T-loop phosphorylation between resting and activated CD4^+^T cells. It is therefore likely that unidentified cellular factors differentially regulate PPM1A or CDK7 enzymatic activity or their sub-cellular localization in resting versus activated CD4^+^T cells. However, as a previous study indicated in PBLs (peripheral blood lymphocytes) as opposed to resting CD4^+^T cells examined in this study, it is possible that under certain conditions, CDK7 protein levels are up-regulated [40]. We had reported that Ca^2+^ signalling is required for CDK9 T-loop phosphorylation [41] and it is possible that a Ca^2+^ influx following T cell activation might modulate PPM1A activity. This implies that in resting CD4^+^T cells, an equilibrium exists between the T-loop phosphorylating mechanism (possibly an upstream activating kinase) and phosphatase activity to meet the low metabolic requirements of quiescent cells. However, in terms of CDK9 T-loop phosphorylation in resting CD4^+^T cells, it appears that the balance is tipped in favor of PPM1A while CDK9 T-loop phosphorylation activity is limited. Upon activation of resting CD4^+^T cells, it is possible that T-loop phosphorylation of CDK9 is up-regulated to meet the transcriptional needs of the cells and this dominates over PPM1A activity.

## Conclusion

In conclusion, our study has highlighted the role of phosphatase PPM1A in regulating CDK9 T-loop phosphorylation and its subsequent effect on HIV-1 replication. It is becoming increasingly evident based on emerging studies that P-TEFb activity in the cell is regulated through its association with host effector proteins. Therefore, the discovery and characterization of regulatory proteins such as PPM1A that modulate P-TEFb activity is important not only for understanding the regulation of cellular gene expression and HIV-1 transcription, but also for developing alternative treatment strategies to perhaps purge the latent viral reservoir and counter HIV-1 resistance to current anti-HIV drugs.

## Methods

### Isolation of primary CD4^+^T cells

Resting primary CD4^+^T cells were purified from buffy coats of healthy anonymous blood donors (Gulf Coast Regional Blood Center, Houston, TX) using RosetteSep (Stem Cell Technologies) which contains an antibody cocktail directed against human hematopoietic cell surface antigens and glycophorin A on erythrocytes. Unwanted cells in the buffy coat are depleted by crosslinking erythrocytes, leading to immunorosettes that are pelleted to obtain the enriched CD4^+^T cells. Resting CD4^+^T cells are isolated from the enriched population of CD4^+^T cells by depleting activated CD4^+^T cells with CD30 microbeads (Miltenyi Biotec). We routinely recovered resting CD4^+^T cell populations that are 98% pure as determined by flow cytometry (data not shown). Resting CD4^+^T cells was activated with anti-CD3/CD28 Dynabeads T-cell expander (Invitrogen) at a ratio of 1:1 (bead/CD4^+^T cell).

### Cell extracts, immunoblots and immunoprecipitations

All cell extracts were prepared at the indicated time points in EBCD buffer (50 mM Tris–HCl [pH 8.0], 120 mM NaCl, 0.5% NP-40, 5 mM dithiothreitol) containing a protease inhibitor cocktail (Sigma). Protein concentrations of cell lysates were measured by the Bio-Rad protein assay which is based on the Bradford method. For detection of proteins in cell lysates, total protein was separated by SDS-PAGE, transferred to nitrocellulose membrane and probed with indicated antibodies. Protein signals were detected using Enhanced Chemiluminescence (ECL) Western Blotting Substrate (Pierce). Immunoblots were analyzed using ImageJ software (NIH).

PPM1A monoclonal antibody (ab 14824) and Phosphorylated CDK7 antibody (ab 59987) was purchased from Abcam. CDK9 (sc-484), α-Tubulin (sc-5286) and CDK7 (sc-529) antibodies were purchased from Santa Cruz Biotechnology. Phosphorylated CDK9 antibody (#2549) was purchased from Cell Signaling Technology.

### HIV-1 pseudotyped virus generation and infection

To produce pseudotyped HIV-1 virus encoding the luciferase gene, HEK 293T cells were co-transfected using Lipofectamine 2000 with plasmids encoding the VSV-G glycoprotein and the HIV-1 NL4-3 virus encoding the Luciferase gene in place of Nef and deleted in Env (pNL4-3.Luc.R-E-; AIDS Research and Reference Reagent Program, NIH). At 48 hours post-transfection, cellular supernatants were collected and the pseudotyped virus was concentrated with PEG-*it*^TM^ virus precipitation solution (System Biosciences) according to the manufacturer’s instructions. Briefly, virus-containing supernatant was incubated with the PEG solution overnight at 4°C, followed by centrifugation at 15,000 G for 30 minutes at 4°C. The pelleted virus was resuspended in PBS, and stored at −80°C. Viral infection of HeLa cells was performed by incubating the purified virus with the growth medium for 5 hours at 37°C. Unbound virus was removed by washing with PBS twice and cultures were incubated overnight at 37°C.

### siRNA and plasmid nucleofection

For siRNA nucleofections, resting CD4^+^T cells were nucleofected on two consecutive days with 200 pmol of siRNA and 100 pmol of siRNA respectively using Amaxa Human T cell Nucleofector Kit (VPA-1002), program U14 according to manufacturer’s directions. Cell lysates were obtained 48 hours post-transfection for immunoblot analysis. For siRNA and plasmid co-nucleofections, resting CD4^+^T cells were nucleofected on two consecutive days with 200 pmol of siRNA and 100 pmol of siRNA plus 5 μg of pNL4-3GFP or 2.5 μg pNL4-3 Luciferase respectively. Cells were analyzed 48 hours post-infection for GFP expression by flow cytometry or Luciferase expression.

### Flow cytometry

GFP expression of each sample was detected by analyzing 10,000 events on BD Canto or BD Fortressa flow cytometers. Data analysis was done using BD FACS Diva software.

### Luciferase and CAT reporter assay

All transfections for Luciferase assays were performed using Lipofectamine 2000 (Invitrogen) in HeLa cells grown in 24 well plates. Cell lysates for both CAT reporter and Luciferase assays were prepared in Reporter Lysis Buffer (Promega). For Luciferase assays with full-length proviral reporter plasmid, pNL43-Luc (500 ng) was cotransfected with empty vector and PPM1A WT (500 ng) or PPM1A MT (500 ng) as indicated. Luciferase assays were performed using the Luciferase Assay System from Promega. Relative Light Units were normalized to total protein in cell lysates.

For CAT reporter assays, HeLa cells were transfected with pSLIIB-CAT reporter plasmid (500 ng), and expression plasmids for effector proteins, Rev-CDK9 (500 ng) or Rev-CDK8 (500 ng), PPM1A WT (50 ng) or PPMIA MT (50 ng), Rev-Tat (500 ng) and Rev (500 ng) as indicated. CAT assays were performed with the Promega CAT Assay Enzyme System according to manufacturer’s instructions. CAT activity was quantified by Liquid Scintillation Counting and normalized to total protein in cell extracts.

### Cell lines, plasmids and reagents

HeLa cells and resting CD4^+^T cells were maintained in DMEM (Invitrogen) and RPMI (Invitrogen), respectively, supplemented with 10% fetal bovine serum (FBS).

Expression plasmids for Flag tagged PPM1A WT, Rev-fusion chimeras, and pSLIIB-CAT have been described previously [25,32]. Flag tagged PPM1A R174G mutant was created using Agilent Technologies QuikChange II Site-Directed Mutagenesis kit according to manufacturer’s instructions. HIV-1 proviral plasmids pNL4-3 Luciferase and pNL4-3 GFP were obtained from NIH AIDS Research and Reference Reagent Program, Division of AIDS, NIAID, NIH.

## Competing interests

The authors declare that they have no competing interests.

## Authors’ contributions

SB performed experiments and analyzed data. RR performed the experiment shown in Figure 5B. AR conceived the study and participated in its design. AR and SB wrote the manuscript. All authors read and approved the final manuscript.
